# Space partitioning in wild, non-territorial mountain gorillas: the impact of food and neighbours

**DOI:** 10.1098/rsos.170720

**Published:** 2017-11-29

**Authors:** Nicole Seiler, Christophe Boesch, Roger Mundry, Colleen Stephens, Martha M. Robbins

**Affiliations:** 1 Department of Primatology, Deutscher Platz 6, 04103 Leipzig, Germany; 2 Max Planck Institute for Evolutionary Anthropology, Deutscher Platz 6, 04103 Leipzig, Germany

**Keywords:** *Gorilla beringei beringei*, space partitioning, home range overlap, movement decisions, intraspecific competition, territoriality

## Abstract

In territorial species, the distribution of neighbours and food abundance play a crucial role in space use patterns but less is known about how and when neighbours use shared areas in non-territorial species. We investigated space partitioning in 10 groups of wild, non-territorial mountain gorillas (*Gorilla beringei beringei*). Using location data, we examined factors influencing daily movement decisions and calculated the per cent overlap of annual kernel home ranges and core areas among neighbours. We found that the probability that a group chose an area was positively influenced by both food availability and the previous use of that area by the group. Additionally, groups reduced their overall utilization of areas previously used by neighbouring groups. Lastly, groups used their core areas more exclusively than their home ranges. In sum, our results show that both foraging needs and avoidance of competition with neighbours determined the gorillas' daily movement decisions, which presumably lead to largely mutually exclusive core areas. Our research suggests that non-territorial species actively avoid neighbours to maintain core area exclusivity. Together, these findings contribute to our understanding of the costs and benefits of non-territoriality.

## 1. Introduction

Access to critical resources is a major determinant of fitness and is influenced by space use patterns [1]. In territorial animals, owners actively exclude conspecifics from fixed areas [2]. Space use patterns are therefore largely determined by between-group competition [3–5] and peripheral areas of the territory are often underused [6–8]. In non-territorial species, individuals or groups do not actively exclude conspecifics from their home ranges [2,9] and less is known about how and when neighbours use shared space (but see [10,11]).

A home range is the overall area used by a group and results from sequential daily movement decisions, reflecting the multi-faceted interactions between an animal's behaviour and its ecological and social environment [12,13]. Movement decisions, i.e. where to move to and how much to use a chosen area, are influenced by both resource availability [14,15] and competition among neighbouring groups [6,8]. Animals may use an area exclusively, but exclusive use and territoriality are not the same [2]. Territories arise when individuals or groups aggressively defend and exclude conspecifics from either part or all the home range containing limited resources [2,9]. Territoriality is adaptive when the benefits of monopolizing limited resources outweigh the costs of excluding conspecifics [16]. Territorial behaviour becomes cost-effective when limited resources within a sufficiently small area can be economically defended against competitors [17–19]. Hence, resource availability and the distribution of neighbours should affect the costs and benefits of sharing space.

Food availability is a crucial factor influencing space use patterns (e.g. [10,20]). According to the optimal foraging theory, animals are predicted to spend more time in areas that yield the highest average rate of energy intake [14,15] and choose habitat types with high-quality food resources (e.g. [21–23]). Animals with overlapping home ranges need to adjust their spacing patterns to maximize foraging efficiency, but at the same time, they need to minimize negative effects of between-group competition.

Sharing space in non-territorial species is influenced by between-group scramble competition, where resources are exploited by the group that arrives first [24,25]. This can lead to increased energetic costs due to reduced food availability and predictability as a result of depletion by neighbours [26,27]. Sharing space also increases the risk of encountering neighbours and possible injury from fighting [6,8], resulting in avoidance-based spacing patterns [10,28]. The number of encounters between neighbours might be expected to increase as home range overlap increases [29,30], although encounters can be rare in cases of high home range overlap [10,31]. Many studies describe encounters between groups (e.g. [32,33]), yet only a few investigated how intraspecific competition results in large-scale patterns of space partitioning [10,31].

To better understand how and when non-territorial neighbours use shared areas, we investigated the impact of both food availability and neighbours on space partitioning in mountain gorillas. Mountain gorillas are ideal for investigating how neighbouring groups share space because they are non-territorial (i.e. they do not actively exclude conspecifics from their home ranges [9]) and show extensive intergroup home range overlap (range of overlap: 13–100%) [29,34,35]. Additionally, they face spatial variation in food availability, despite little seasonal variability [36,37] and between-group competition for access to mates [38,39]. Mountain gorillas live in stable and cohesive social units (mean group size: 10, range: 2–47), containing one or more adult males, several adult females and their dependent offspring [40–42]. Although gorillas adapt their space use to the spatial variation in food availability [35,37,43], the importance of food in influencing interrelated movements among neighbours remains unknown.

Intraspecific competition in gorillas has been attributed mainly to mate competition, with females transferring between neighbouring groups during intergroup encounters [38,44]. However, despite large intergroup home range overlap [34], encounters between groups are rare (monthly average: 0.78 [39]). Encounters impact moving patterns by increasing daily travel distances on the days of encounters [45]. In addition, the local gorilla population density has a negative relationship with monthly home range size, suggesting that groups contract their ranges as intraspecific competition increases [45].

The main objectives of this study were (i) to evaluate how groups adapt their spacing pattern to food availability and neighbours' use patterns on small temporal and spatial scales (i.e. daily) and (ii) to examine how this relates to the usage of shared space among neighbouring groups on a larger scale (i.e. annual). We predicted that gorilla groups will adjust their daily movement decisions to the spatial variability of herbaceous food resources by foraging in areas with high food availability. At the same time, we predicted that groups will adapt their movement decisions to both their own and to the neighbours’ previous ranging patterns. Specifically, we predicted that gorillas will use those areas with (i) higher herbaceous food availability, (ii) lower previous use by the group, and (iii) lower previous use by neighbouring groups. However, Virunga gorillas do not deplete previously used areas [43] and therefore, we alternatively predicted that gorillas will use those areas with (iv) higher previous use by the group. Lastly, examining space partitioning among neighbours on a large scale, we predicted that annual core areas will be more exclusive (i.e. less overlap among neighbours) than annual home ranges.

## 2. Material and methods

### 2.1. Study site and data collection

**Figure 1.**
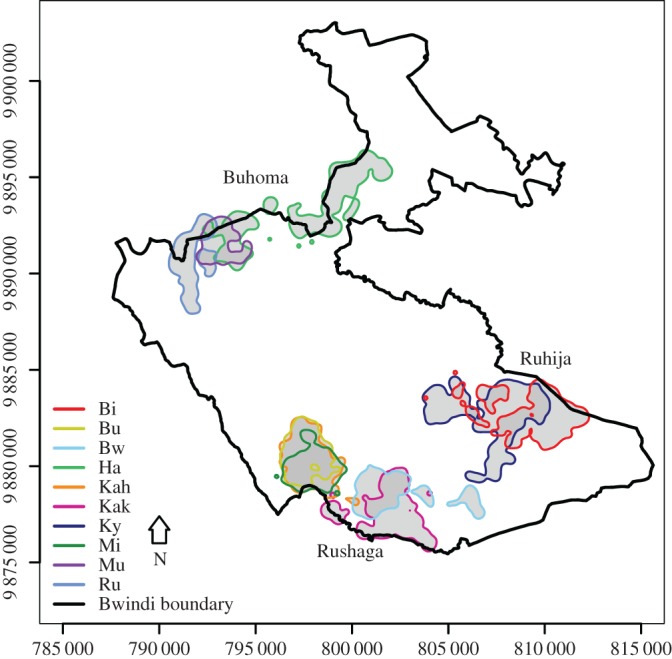
Annual kernel home ranges of the 10 mountain gorilla groups studied in 2012 and 2013 in Bwindi Impenetrable National Park, Uganda. Home range areas (90% fixed kernel density estimates) in the three general locations of the study groups are depicted in grey and shared areas are indicated by darker grey shading.

We studied 10 habituated groups of mountain gorillas in Bwindi Impenetrable National Park, Uganda, between May 2012 and July 2013, which represent nearly one-third of the social units and individual gorillas in the population [41]. All study groups shared their annual 90% kernel home range with at least one neighbouring group (figure 1). We collected all-day location data using global positioning system (GPS) units (GPSmap 60CSx and 62) recording in 30 s intervals. Part of the all-day location data were recorded by following the groups during the direct observation time, which was limited to 4 h d^−1^ by park authority regulations to reduce anthropogenic disturbance. Owing to the restriction of direct observation time, we collected the rest of the data by walking along the main trail that was left by a gorilla group on a respective day. Following the trails gave data on distance travelled in an area but not temporal use of an area. These trails are easily detectable by bent vegetation, dung and food remains [37,46]. Data were collected by N.S. and trained field assistants for an average of 13 months per group (range: 8–14 months) and for an average of 16 days (range: 2–31 days) per month and group (for more details, see [45]). The number of observation months was less than 1 year for two groups (Bw and Kak) because data collection on them began following a group fission during the study period. On a bi-monthly basis, N.S. checked the compliance of each assistant with the data collection protocol. We determined the mean group size per month for each group, defined as the average number of weaned individuals (range: 4–13 individuals) because group composition changed for some groups by one to two individuals during the study period.

### 2.2. Movement decisions

#### 2.2.1. Herbaceous food availability per grid cell

Herbaceous food availability per grid cell was measured as the energy density of herb species that contributed to at least 1% of the diet recorded for all groups over the study period (*N* = 24) [36]. Those species were determined based on instantaneous scan sampling at 5 min intervals of all weaned individuals in view throughout the daily observation period to record dietary intake of the study groups. We directly observed 124 gorillas for an average total observation time of 258 h (range 86–1383 h).

Energy density was based on biomass density estimates derived from vegetation sampling and nutritional content of herb species. As the temporal variation of herbaceous biomass in Bwindi is negligible [36], we determined biomass of herbaceous food species once by sampling a total of 490 transects. Transects were of 200 m length and randomly placed within 500 × 500 m grid cells (for details and justification, see electronic supplementary material, S1) overlaid onto a map including the study groups' home ranges. For each plant and along each transect, we measured stem length or number of leaves in 10 1 m^2^ plots, which were placed on alternate sides in 20 m intervals [36,46]. Using regression equations relating the respective measure recorded in the vegetation transects to the dry weight of sampled plants (electronic supplementary material, S2 and table S2), we calculated dry biomass (g m^−2^). We then multiplied the predicted metabolic energy of each herb species (kcal g^−1^) estimated from nutritional analysis by its biomass (g m^−2^) and summed all species’ energy contents (kcal m^−2^) to determine energy density per grid cell (for more details, see electronic supplementary material, S2). The average energy density of the most important herbaceous food species per grid cell was 959 kcal m^−2^ (range: 0–13 054 kcal m^−2^; electronic supplementary material, S2 and figure S2).

#### 2.2.2. Previous use of grid cells by the group and by its neighbouring groups

We used all-day location data for each group (location points taken every 30 s) to calculate (i) the previous use of grid cells by the group and (ii) the previous use of grid cells by its neighbouring groups. For each day and each group, we determined the previous use of all visits to each 500 × 500 m grid cell by both the group and also by all habituated neighbouring groups collectively. This resulted in two predictor variables per group and day: the previous use by the group and the previous use by its neighbouring groups. We incorporated three measures into these two variables: (i) mean group size, to account for larger groups depleting an area more than smaller groups, (ii) time since the previous visit to a given cell, as a measure of time for regeneration of food resources, and (iii) distance travelled during the previous visit, as a proxy for the utilization of that cell (for details and justification, see electronic supplementary material, S3). As a composite measure of previous use for each group and visit to a grid cell, we divided the size of the group and each neighbouring group, respectively, by the time passed in days since the last visit of that group to a cell and multiplied this term by the distance travelled by that group in that cell during that visit. We then summed up each estimate for each previous visit by the group and by its neighbouring groups per group and grid cell:

$$\text{Previous use}=\sum_{i=1}^{n\;\mathrm{visits}}\left[\left(\frac{\text{group size}}{\text{time passed since last visit}}\right)\times \text{length of track during the last visit}\right].$$

Although the Bwindi census 2011 identified 26 unhabituated groups (range of group sizes: 2–17) [41], we were not able to include them in the analysis because we only had on average 3.1 location points (range: 1–11) per group and hence could only estimate their approximate home ranges (see electronic supplementary material, S4). During the 2011 census, home range centres of only three of the 26 groups (group sizes: 3, 9 and 17) were found in the periphery of the 2012–2013 annual home ranges of some study groups but none in the study groups' core areas (electronic supplementary material, S4 and figure S4*a*,*b*). Therefore, we assume that excluding the unhabituated groups did not bias our results. Furthermore, our variables might underestimate previous use by groups and by neighbouring groups because we cannot assess the use of grid cells on days when we did not collect data. However, we collected data on approximately 50% of all days during the study period for most groups (table 1; except for groups Bw and Kak, which formed following a group fission) and therefore, we assume the overall patterns to be representative. Because we did not have data about home range use prior to the start of the study, we determined a point in time when sufficient data on previous use were available. Based on visual inspection of plots showing the predictor variable plotted against date (electronic supplementary material, S5 and figure S5*a*,*b*), we decided 1 October 2012 (five months into the study) as appropriate and restricted analyses to data collected from that day onward.

**Table 1.** Annual kernel home range sizes of the Bwindi mountain gorilla study groups and per cent of area overlap of a group with all habituated neighbouring groups. Home range sizes and overlap estimates are shown for annual home ranges (90% kernel home range) and annual core areas (50% kernel home range). Overlap estimates range from zero (=no overlap) to 100 (=100% overlap). The number of location data points used for annual home range and core area estimates corresponds to the number of observation days per group. The high home range and core area overlap of the groups Busingye, Mishaya and Kahungye may be due to two group fissions during the study period.

group	annual kernel home range (km^2^)	annual kernel core area (km^2^)	exclusively used part of core area (km^2^)	per cent overlap of annual home range	per cent overlap of annual core area	no. location data points used for annual analysis
Bitukura (Bi)	12.03	3.41	3.11	43.45	8.62	162
Kyagurilo (Ky)	15.01	4.46	4.17	34.83	6.59	394
Busingye (Bu)	7.37	2.33	0.88	94.79	62.09	204
Mishaya (Mi)	6.42	1.94	0.62	91.69	67.87	202
Kahungye (Kah)	8.90	3.15	0.75	93.24	76.09	191
Bweza (Bw)	7.66	2.51	2.47	40.60	1.93	128
Kakono (Kak)	9.90	3.49	3.44	30.95	1.39	105
Mubare (Mu)	4.50	1.10	1.10	65.52	0.05	205
Habinjanya (Ha)	14.01	3.88	3.88	17.18	0.01	195
Rushegura (Ru)	6.39	1.47	1.47	9.68	0	198

#### 2.2.3. Probability of choosing a particular area

For each group and each decision to move into another 500 × 500 m grid cell, we determined which of the eight surrounding cells was entered (electronic supplementary material, figure S6) based on all-day location data. The cell entered was assigned a one; the non-chosen cells were each assigned a zero. We counted multiple entries into the same grid cell on the same day as one decision.

#### 2.2.4. Utilization of a chosen area

We determined the distance travelled by each group in each 500 × 500 m grid cell using all-day location data. We used this as a proxy for the utilization of a chosen area because we could not assess the gorillas’ temporal use when following only their trails, but we assumed a positive relation between the distance travelled and the area used. Using this proxy is justified because mountain gorillas spend about 50% of their day feeding and spend only little time exclusively travelling to search for food [47]. Additionally, we investigated activity patterns of one study group (Ky) using instantaneous scan sampling at 5 min intervals of the group's activity (*N* = 18 459 scans) recorded over the study period, which further affirmed that distance travelled is a good proxy for utilization (electronic supplementary material, S3 and figure S3).

#### 2.2.5. Statistical analysis: probability of choosing a particular area

To investigate which factors influenced the probability that a group would choose a particular area (i.e. grid cell), we developed a generalized linear mixed model [48] (*N* = 11 811 observations from 10 groups) with binomial error structure (binary response variable: grid cell chosen yes/no) and logit link function [49]. As test predictors we included (i) herbaceous food availability of the respective grid cell, (ii) the previous use of that cell by the group, and (iii) the previous use of that cell by neighbouring groups. We log-transformed all test predictors to achieve approximately symmetrical distributions and then *z*-transformed them to a mean of zero and a standard deviation of one [50]. The inverse of the number of surrounding grid cells was included as an offset-term (log-transformed) to control for the varying number of surrounding cells (usually eight, but in some cases, the number varied because some groups ranged on the edge of the park and food availability data were missing). To control for repeated observations, we included group ID (*N* = 10), an ID for the possible grid cells into which a group could move (grid cell ID; *N* *=* 341), an ID for the group of the surrounding grid cells, reflecting the choices a group had (choice ID; *N* *=* 1777), and an ID for the possible grid cells in which a group could move, nested within group (group-grid cell ID; *N* = 601). We included choice ID to account for the non-independence of choices as each time only one of the surrounding cells can be chosen. Additionally, we accounted for group-grid cell ID because the same groups might have had preferences for particular grid cells and hence repeatedly moved to the same cells. To keep error I rate at the nominal level of 5%, we included random slopes where applicable (electronic supplementary material, S7) [51,52]. We conducted permutations for each choice to move to another grid cell, which randomly shuffled the assigned one from the actual chosen grid cell among all surrounding cells [53,54]. As the probability that the gorillas would choose one of the four directly adjacent cells was higher than the probability that they would choose a cell bordering the corner of the cell of origin (see electronic supplementary material, figure S6), we adjusted the probabilities for a particular cell to be randomly chosen correspondingly. We could not control for the possibility that gorillas on the edge of a grid cell may be more likely to move to the cell adjacent to the respective edge because we could not quantify this probability. For details of model implementation and R syntax of the fitted model, see electronic supplementary material, S7.

#### 2.2.6. Statistical analysis: utilization of a chosen area

To investigate which factors influenced the utilization of a chosen area (i.e. grid cell; *N* = 3378 observations from 10 groups), we used a linear mixed model and fitted it with Gaussian error structure and identity link [48]. As test predictors, we included (i) herbaceous food availability of the respective grid cell, (ii) the previous use of that cell by the group, and (iii) the previous use of that cell by neighbouring groups. All test predictors were log- and then *z*-transformed [50]. We included test predictors centred to a mean of zero per group (=within-groups variation) and the mean of the predictors per group (=between-groups variation) to account for possibly different effects of the predictors within and between groups [55] (electronic supplementary material, S7). As random effects, we included group ID (*N* = 10), grid cell ID (*N* = 266) and group-grid cell ID (*N* = 422). We included an autocorrelation term derived analogously to that described by Fürtbauer *et al*. [56] (electronic supplementary material, S7). We included the random slopes of the within-groups effects where applicable (electronic supplementary material, S7) [51,52]. For details of model implementation and R syntax of the fitted model, see electronic supplementary material, S7.

### 2.3. Annual home range overlap

#### 2.3.1. Per cent overlap of annual home ranges and core areas

Applying fixed kernel density estimation [57], we calculated annual home ranges (90% kernel home range) and core areas (50% kernel home range; following the recommendations by Börger *et al*. [58]) per group using the adehabitatHR package [59] in R [60]. This method generates utilization distributions, which represent groups' relative use of space [61]. The kernel density estimate is a method based on point densities [57], but we could not assess the groups’ temporal use when following the trails, and hence we restricted the home range and core area estimation to one location point per day per group (first recorded location per day). Choosing the first recorded location per day gives a representative space use estimate as gorillas only move on average 808 m d^−1^ (range: 547–1034 m) between two consecutive night nest sites [34], which are constructed every night at a different location [41]. We used on average 198 location data points per group (range: 105–394; table 1) to estimate annual kernel home ranges and fixed the bandwidth to *h* *=* 200 [29]. This allowed us to produce home range contours for all groups with relatively little fragmentation. Using sensitivity analyses comparing home range sizes using subsamples, we showed that the chosen parameter (*h* = 200) revealed rather robust estimates (electronic supplementary material, S8 and figure S8).

For each group, we calculated the sizes of areas shared with neighbouring groups and estimated both: (i) the per cent of the annual home range covered by the home ranges of all other habituated neighbouring groups; and (ii) the per cent of the annual core area covered by the core areas of all other habituated neighbouring groups (table 1). Using the polygons forming the 50% and 90% kernel home ranges, we determined the polygons of the shared areas. The per cent home range overlap of group *i* was quantified as follows:

$$\begin{array}{rl} & \text{Per cent home range overlap}:\;100\times \frac{{\text{SH}}_{i}}{H_{i}},\\ & \text{Per cent core area overlap}:\;100\times \frac{{\text{SC}}_{i}}{C_{i}}, \end{array}$$

where SH*_i_* is the size of the area shared of the home range and SC*_i_* the size of the area shared of the core area of group *i* with all other habituated neighbouring groups, *H_i_* the home range size and *C_i_* the core area size of group *i* (for proportions of dyadic annual home range and core area overlap, see electronic supplementary material, S9 and table S9). Unhabituated groups could not be included, but we assume that excluding them did not bias our results (see electronic supplementary material, S4).

#### 2.3.2. Statistical analysis of annual home range and core area overlap estimates

We used a non-parametric Wilcoxon signed-ranks test [62] to compare the per cent of area overlap of the annual home ranges with the per cent of area overlap of the annual core areas. Tests were exact [62,63] and were calculated using the package exactRankTests [64] in R [60]. All *p*-values are two-tailed.

Additionally, as a *post hoc* test, we examined the prediction that core areas had higher herbaceous food availability than the rest of the home ranges using a non-parametric Wilcoxon signed-ranks test [62]. To do so, we determined herbaceous food availability of core areas and the rest of the ranges. We based our measure of herbaceous food availability on the energy density (kcal m^−2^) per 500 × 500 m grid cell (see *Herbaceous food availability per grid cell*) and used the polygons forming the 50% and 90% kernel home ranges. Herbaceous energy density was calculated by summing the herbaceous energy density of all grid cells encompassed in an area (i.e. core area and home range). As most grid cells were encompassed to various extents in an area, the summed herbaceous energy density was weighted by the size of the overlap of each area with each grid cell and divided by the size of an area. We used the packages spatstat [65], splancs [66] and SDMTools [67] in R [60] for processing and analysing spatial data.

## 3. Results

### 3.1. Movement decisions

#### 3.1.1. Probability of choosing a particular area

**Figure 2.**
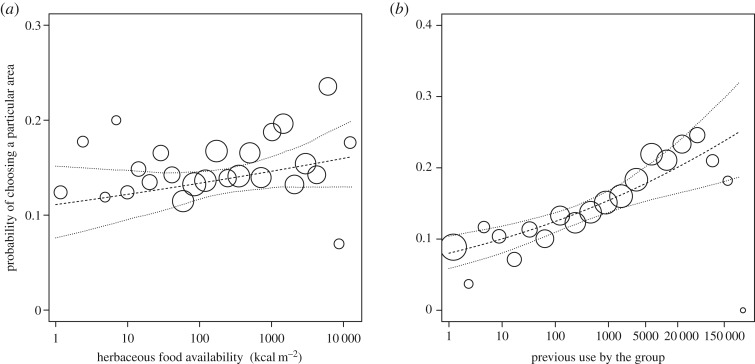
Influence of (*a*) herbaceous food availability (kcal m^−2^, based on herb biomass and nutritional content) and (*b*) previous use by the group on the probability of choosing a particular area (i.e. a 500 × 500 m grid cell) in Bwindi gorillas. The area of the circles indicates the fourth root of the number of observations. In (*a*), the largest circle corresponds to 1268 and the smallest circle corresponds to 30 observations, whereas in (*b*), the largest circle corresponds to 2111 and the smallest circle corresponds to three observations. The dashed and dotted lines indicate the fitted influence of the predictor on the response and its confidence intervals, respectively, with all other predictor variables in the model being at their average.

When investigating the factors influencing the probability that a group would choose a particular area (i.e. the decision which of the eight surrounding cells to move to), we found a significant effect of the test predictors as a whole (full null model comparison, permutation test: *χ*^2^ = 19.228, d.f. = 3, *p* = 0.003). As predicted, we found that the probability that a group chose a particular area was positively influenced by the availability of herbaceous food of that area (figure 2*a*). Furthermore, areas were chosen more frequently when the previous use of that area by the group increased (figure 2*b*). The previous use by neighbouring groups did not have an apparent effect (table 2).

**Table 2.** Summary of the permutation test and the mixed model results investigating the factors influencing the probability that Bwindi mountain gorilla groups would choose a particular area (i.e. grid cell) and the utilization of a chosen area (quantified as distance travelled per grid cell). For each model, we show the *χ*^2^ value, degrees of freedom (d.f.) and the *p*-value of the full null model comparison. We show the estimate (Est), standard error (s.e.) and *p*-value for each test and control predictor; (within) indicates the within-groups effect and (between) indicates the between-groups effect of a predictor variable. The autocorrelation term (Autocor) represents temporal and spatial autocorrelation. Empty cells indicate variables not included in a model. For reasons of completeness, we present the results of the random effects in the electronic supplementary material, table S10. Mean and standard deviation of the original values of the predictor variables are shown in the electronic supplementary material, table S11. Significant results (*p* < 0.05) are indicated in bold.

response variable	probability of choosing a particular area			utilization of a chosen area			
full null model comparison	*χ*^2^ = 19.228, d.f. = 3, *p* *=* 0.003			*χ*^2^ = 22.341, d.f. = 6, *p* *=* 0.001			
predictor variable	Est	s.e.	*p*-value	Est	s.e.	*χ*^2^	*p*-value
intercept	0.072	0.036	^a^	5.970	0.032	^a^	^a^
herbaceous food availability (within)	^b^			0.000	0.028	0.000	0.994
herbaceous food availability (between)	**0.080**	**0.039**	**0.002**	−0.040	0.036	1.105	0.293
previous use by the group (within)	^b^			0.084	0.036	3.753	0.053
previous use by the group (between)	**0.354**	**0.059**	**0.001**	0.076	0.036	3.512	0.061
previous use by the neighbours (within)	^b^			−0.032	0.039	0.637	0.425
previous use by the neighbours (between)	0.002	0.039	0.898	**−0.161**	**0.036**	**11.858**	**<0.001**
Autocor				0.255	0.047	14.329	<0.001

^a^Not shown because of having no meaningful or a very limited interpretation.

^b^There were no within-groups effects for this model.

#### 3.1.2. Utilization of a chosen area

**Figure 3.**
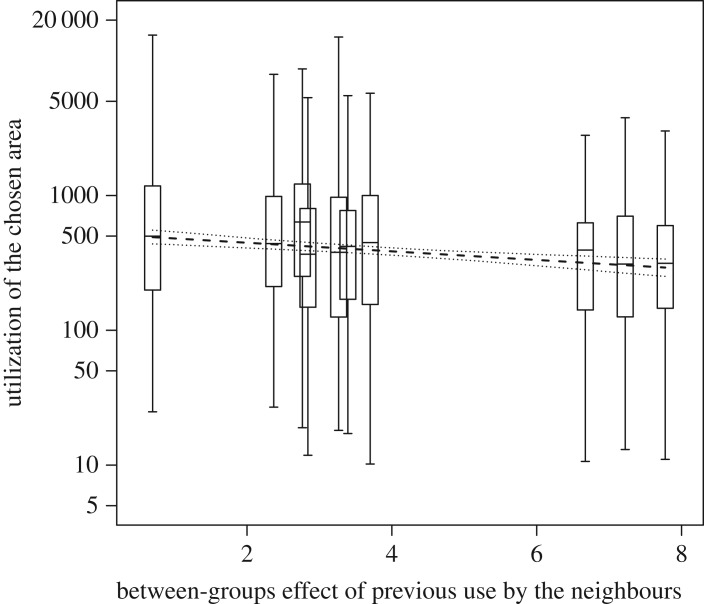
Influence of the between-groups effect of previous use by all habituated neighbouring groups on the utilization of a chosen area (quantified as the distance travelled in a chosen 500 × 500 m grid cell) in Bwindi gorillas. The response variable was log-transformed. Between-groups variation is expressed as the mean of the previous use per group. Boxes depict quartiles with the median values indicated as horizontal lines and vertical lines show quantiles (2.5 and 97.5%). The dashed line indicates the fitted influence of the predictor on the response, with all other predictor variables in the model being at their average. The dotted lines depict bootstrapped 95% confidence intervals of the model.

We found a significant effect of the test predictors as a whole on the utilization of a chosen area (likelihood ratio test: *χ*^2^ = 22.341, d.f. = 6, *p* = 0.001). The average utilization of areas by groups decreased as the average use of the same areas by neighbouring groups increased (between-groups effect; figure 3). We found a tendency for a positive effect of the previous use of an area by the group for both the within- and the between-groups effect on the utilization of a chosen area. Food abundance did not have a clear effect (table 2).

### 3.2. Annual home range and core area overlap

**Figure 4.**
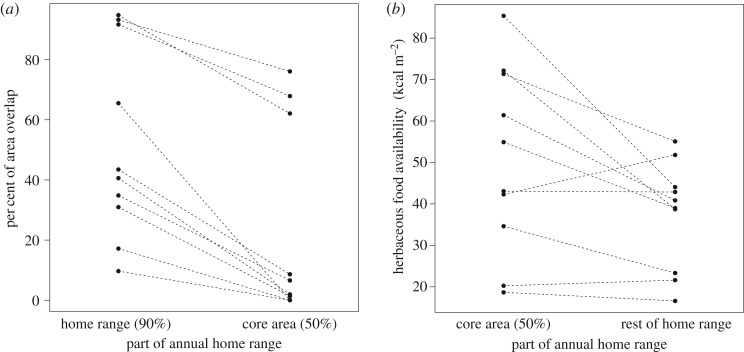
Annual home range and core area overlap of the Bwindi gorilla groups and herbaceous food availability of their core areas and the rest of their respective home ranges. (*a*) Per cent overlap of annual home ranges (90% kernel home range) and core areas (50% kernel home range). (*b*) Herbaceous food availability (kcal m^−2^, based on herb biomass and nutritional content) of core areas (50% kernel home range) compared to the rest of the respective home ranges. Dashed lines connect data points from the same respective group. The high home range and core area overlap of three groups (Bu, Mi and Kah) may be due to two group fissions during the study period.

We found that groups shared a significantly lower percentage of their annual core area than their annual home range with their neighbours (Wilcoxon signed-ranks test: *T^+^* = 55, *N* = 10, *p* = 0.002; figure 4*a*). The median percentage of annual home range overlap was 42.02% (range 9.68–94.79%) and the median percentage of annual core area overlap was 4.26% (range 0–76.09%; table 1). Three groups (groups Bu, Mi and Kah; figure 1 and table 1) had high core area overlap. As gorilla groups that form following group fissions may remain in the same areas as the original groups for at least 1 year [29], social factors seem to have caused this high degree of overlap (Bu fissioned from Kah, and both Bw and Kak were the result of a fission that might have affected Mi). Lastly, core areas were characterized by significantly higher herbaceous food availability than the rest of the home ranges (*T^+^* = 6, *N* = 10, *p* = 0.027; figure 4*b*).

## 4. Discussion

Our study sheds new insights into how intraspecific competition and food availability influenced space partitioning in a non-territorial species, the mountain gorilla, on two scales: daily movement decisions and overlap of annual home ranges and core areas. We found that core areas of neighbouring groups were more mutually exclusive than their home ranges. Based on seven of 10 groups having core area overlap of less than 10%, we suggest that Bwindi mountain gorillas have largely mutually exclusive core areas. This is a novel finding for this non-territorial species, known to exhibit large intergroup home range overlap [29,34,35]. The maintenance of such a pattern without territorial defence may result from the gorillas' decision to return repeatedly to areas with high food availability and to reduce using areas previously used by neighbours. These behavioural patterns may be due to three mechanisms (see below) to actively avoid shared areas.

### 4.1. Ecological factors

Our results suggest that Bwindi gorillas returned repeatedly to areas of high food availability, resulting in annual core areas that were of higher quality than the rest of the ranges. Similarly, Virunga gorillas stay relatively long per visit in areas with abundant food and return to them often at relatively short intervals [43]. Repeatedly returning to areas of high food availability may trigger and maintain a positive feedback loop in which animals return to areas where they can feed on newly grown food with high nutritional quality and maintain a higher density of food plants within their range [68–70]. Comparable to our results, Virunga gorillas and other species adapt their movements to their foraging needs by choosing high-quality habitat types (e.g. [21,22,35,43]). We found no effect of food availability on the utilization of a chosen area, possibly because the differences in herbaceous food availability between the chosen high-quality areas were too small to have an impact. Together, these results suggest that mountain gorillas do not deplete particular areas and that they know and remember where to find good foraging areas. Future studies should investigate gorillas’ spatial and temporal knowledge of their habitat and how this affects movement decisions [1,71].

### 4.2. Intraspecific competition

Gorillas appear to actively avoid neighbouring groups on an annual and a daily scale, despite having considerable intergroup home range overlap (table 1). Groups were less likely to use areas previously used by neighbours and annual core areas were largely mutually exclusive. This lends further support to the existence of competition among Bwindi gorilla groups, based on previous results showing that monthly home range size decreases as the local gorilla population density increases [45]. However, against our prediction, we found no significant impact of the previous use by neighbours on the probability that a group chose an area, which suggests that groups typically do not know their neighbours' location.

### 4.3. Mechanisms to avoid shared areas

We suggest three mechanisms by which gorillas may avoid neighbours and maintain largely mutually exclusive core areas without territorial defence: intergroup encounters, chest beats and visual inspection of areas where other groups have foraged. On days when intergroup encounters occur, Bwindi gorillas increase their daily travel distance [45], suggesting that groups might either leave their core areas and then meet neighbouring groups or they retreat to their core areas following an intergroup encounter in the shared parts of their home range (see also [72]). Gorillas may remember the locations of these encounters and subsequently avoid those areas. Encounters between gorilla groups seem to stimulate an avoidance response and hence may function like a spacing mechanism and a ‘keep-out’ signal ([2], see also [73,74]), which are characteristic aspects of territorial behaviour [2].

The non-vocal chest beat, which can be heard from a maximum distance of 500–1000 m [75] (N.S. and M.M.R. 2012, personal observation), may have evolved as an honest signal of strength and fighting ability of males to attract females and repel competitors [76,77]. Used in both within- and between-group communication [78], chest beating might also function as long-distance signalling to locate neighbouring groups. Therefore, chest beats may serve as a spacing mechanism, comparable to loud calls in territorial species (e.g. [79]). When groups are further apart from each other (greater than 1000 m), groups may use signs of foraging to locate areas used by neighbours and avoid those.

One ultimate reason for the observed avoidance behaviour among neighbouring gorilla groups might be male mate defence. Male gorillas may be considered as ‘hired guns’ that protect their females and offspring [80,81]. During intergroup encounters, males herd their females away from extragroup males and engage in aggression with those males to prevent both their mates from transferring to other groups and infanticide of their offspring [38,81]. In Bwindi gorillas, 75% of recorded intergroup encounters are characterized by aggression involving displays and chest beating, though only 2.5% involve physical aggression [39]. Encounters between gorilla groups can lead to home range shifts [73], male mortality [73,82] and female transfers [38,44]. Intergroup encounters may serve as a mechanism to both establish and maintain exclusive areas, whereas chest beats and visual inspection may mainly serve the maintenance of the avoidance-based spacing pattern. Between-group competition for mates, which may result in keeping competitors out of certain areas, may also effectively function to indirectly defend food resources within these areas ([80], see also [83,84]). Future studies should investigate whether larger gorilla groups with a strong dominant male [85,86] have more profitable home ranges than smaller groups [87].

## 5. Conclusion

### 5.1. Patterns and consequences of space partitioning

Exclusive use of an area and territoriality are not the same; exclusive occupancy of an area might be caused by mutual avoidance and not only by active defence of a territory [2]. We propose that non-territorial species, like the mountain gorilla, use some aspects of territorial behaviour to actively avoid neighbours and maintain a spacing pattern of exclusivity, which suggests a gradient between territoriality and non-territoriality. Territoriality and exclusivity change with resource availability (e.g. [17,18,88]), but the costs and benefits of territoriality may also depend on social factors (see also [10,30,74]), such as the level of intraspecific competition for mates. For example, animals feeding on abundant resources and having minimal intraspecific competition among neighbours may exhibit little or no avoidance behaviour and thus may not have exclusive areas within an undefended home range. This would place them at the extreme end of the gradient of non-territoriality. The avoidance-based pattern of shared home ranges but largely mutually exclusive core areas in Bwindi gorillas might be stimulated by strong intraspecific competition for mates despite abundant food resources [36]. This would put gorillas in the middle of a gradient between territoriality and non-territoriality.

Spacing patterns to avoid intraspecific competition with neighbours are likely to have important consequences. Social factors, such as defence of mates, that promote exclusivity may have long-term costs by reducing available space and lowering the carrying capacity of an ecosystem despite abundant food resources (see also [8]). In gorillas, these social constraints may also reduce or prevent depletion of food resources in the shared areas of the home range, thereby resulting in consistently highly abundant food resources and hence no selective pressure to actively defend a home range [18]. Our study suggests that non-territoriality may carry more costs than previously thought when social factors limit the use of shared areas.

## Supplementary Material

Section S1; Section S2; Section S3; Section S4; Section S5; Figure S6; Section S7; Section S8; Section S9; Table S10; Table S11; Section S12

## Data accessibility

Our data are available at Dryad Digital Repository: http://dx.doi.org/10.5061/dryad.6k6f6 [89]. Additional information is included in the electronic supplementary material.
