# Supplementary material for: Space partitioning in wild, non-territorial mountain gorillas: the impact of food and neighbours
Source: R Soc Open Sci. 2017 Nov 29;4(11):170720. doi: 10.1098/rsos.170720 (PMC5717636; doi:10.1098/rsos.170720)
Supplement: Section S1; Section S2; Section S3; Section S4; Section S5; Figure S6; Section S7; Section S8; Section S9; Table S10; Table S11; Section S12 [file rsos170720supp1.docx]

**Supplementary** **Material**

Space partitioning in wild, non-territorial mountain gorillas: the impact of food and neighbours

Nicole Seiler, Christophe Boesch, Roger Mundry, Colleen Stephens, and Martha M. Robbins

Section S1 Justification of the 500 x 500 m grid cell method

Section S2 Herbaceous food availability per grid cell

Section S3 Utilization of an area

Section S4 Unhabituated groups

Section S5 Determination of the date at which sufficient data on previous use were available

Figure S6 Graphical representation of a group’s decision regarding which of the surrounding grid cells to choose

Section S7 Statistical analysis

Section S8 Sensitivity analyses to test the robustness of the chosen bandwidth *h*

Section S9 Proportions of dyadic annual home range and core area overlap

Table S10 Random effects for the permutation test and the mixed model results

Table S11 Mean and standard deviation of the original values of the predictor variables

Section S12 References for Supplementary Material

Section S1 Justification of the 500 x 500 m grid cell method

Our measure of food availability was based on sampling in 500 x 500 m grid cells. We sampled herbaceous gorilla food species in 490 transects to get estimates of herbaceous food availability. These transects were randomly placed within 500 x 500 m grid cells overlaid onto a map including the study groups’ home ranges. The chosen grid cell size was a compromise between spatial resolution and feasibility. However, using the 500 x 500 m grid cell method, we followed a protocol from a previous study investigating the spatial and temporal availability of herbaceous vegetation consumed by gorillas in Bwindi [1]. This study found that the spatial availability of gorilla food species differed significantly among habitat types [1] and hence, such a grid size seems appropriate to reflect spatial variability in herbaceous food availability.

Additionally, a previous study found that the daily travel distance of Bwindi gorillas was on average 808 m (range: 547-1034 m) [2] and hence, a grid cell roughly represents the area used for the daily foraging activities of a group. As we were investigating daily movement decisions, a cell size of 500 x 500 m is appropriate to reflect the daily foraging activities of a group. We based the rest of our analyses on estimates per grid cell because our estimates of gorilla food availability were per 500 x 500 m grid cell. Consequently, the all-day location data for a group within the same cell have the same food availability estimate. Therefore, we decided to do all our analyses based on grid cells.

Section S2 Herbaceous food availability per grid cell

*Biomass regression*

We calculated dry biomass (g/m^2^) using regression equations, which related the respective measure recorded in the vegetation transects to the dry weight of sampled plants. Regression equations for 16 species were known [1] and established for eight additional herb species. To do so, we collected 40 randomly chosen specimens per species and measured the length of the stem or number of leaves, respectively, as well as the wet weight of the part eaten by the gorillas. We then sun-dried the specimens and recorded the dry weight of each sample [1,3]. For the tree fern (*Cyathaea manniana*), we did not cut and process 40 specimens to reduce habitat disturbance on this rare and slowly growing plant. We only collected five samples and measured the dry and wet weight of the part eaten by the gorillas.

To establish the regression equations for each herb species (table S2), we plotted stem length or number of leaves against the dry and the wet weight and calculated a linear regression that was forced through the origin [1,4]. Due to the highly significant relationship between stem length or number of leaves and their corresponding dry weights, our measures can be considered good predictors of biomass. We used dry weights only to establish the regression equations to control for variation in water content of the herbs throughout the year [1,3]. To assess the accuracy of the biomass estimate, we bootstrapped confidence limits of the slope of the regression using accelerated bias-corrected confidence limits [5].

Using the regression equations, we calculated biomass of each sampled herb species in a plot and then summed all species’ biomasses to get biomass density per plot. However, for the tree fern (*C. manniana*), we took an average of the dry weights and multiplied this with the number of specimens in each plot to get a biomass estimate.

Table S2. Biomass estimate and regression relationship for the herbs investigated. For all species *N* = 40. All *p-*values were < 0.001.

| Plant species and part consumed by gorillas | Type of biomass estimate | Regression equation | R^2^ |
| --- | --- | --- | --- |
| *Impatiens sp.* – leaves | Leaf mass/ number of leaves | *y* = 0.03 * *x* | 0.88 |
| *Smilax anceps* – leaves | Leaf mass/ number of leaves | *y* = 0.2 * *x* | 0.81 |
| *Pilea holstii* – leaves | Leaf mass/ number of leaves | *y* = 0.04 * *x* | 0.69 |
| *Momordica pterocarpa* – leaves | Leaf mass/ number of leaves | *y* = 0.13 * *x* | 0.86 |
| *Brillantaisia sp.* – pith | Pith mass/stem length | *y* = 3.56 * *x* | 0.69 |
| *Urera sp.* – leaves and bark | Leaf and bark mass/stem length | *y* = 0.08 * *x* | 0.73 |
| *Laportea sp.* – leaves and bark | Leaf and stem mass/ number of leaves | *y* = 0.71 * *x* | 0.76 |

*Energy density estimates*

Nutritional content was known for 73 gorilla food species (which included 67% of the most important herbaceous food species) [6] and eight additional species were analysed using the same methodology in the same laboratory. Nutrient values were expressed as a percentage of organic matter (OM). For each herb species, we first calculated total non-structural carbohydrates (TNC) following Wright et al. [7] using the formula:

TNC = 100 - %*L* - %*CP* - %*TA* - %*NDF*

where *L* is lipids, *CP* is crude protein, *TA* is total ash, and *NDF* is neutral detergent fibre. We calculated the predicted metabolic energy (PME) per gram of OM [7] as

PME (kcal/g) = [(4 × %*CP*) + (4 × %TNC) + (9 × %*L*) + (1.6 × %*NDF*)]/100.

We used conversion factors for *CP*, *L* and TNC from nutritional studies of adult humans and corrected for the use of available energy by anaerobic bacteria for digestions [7].


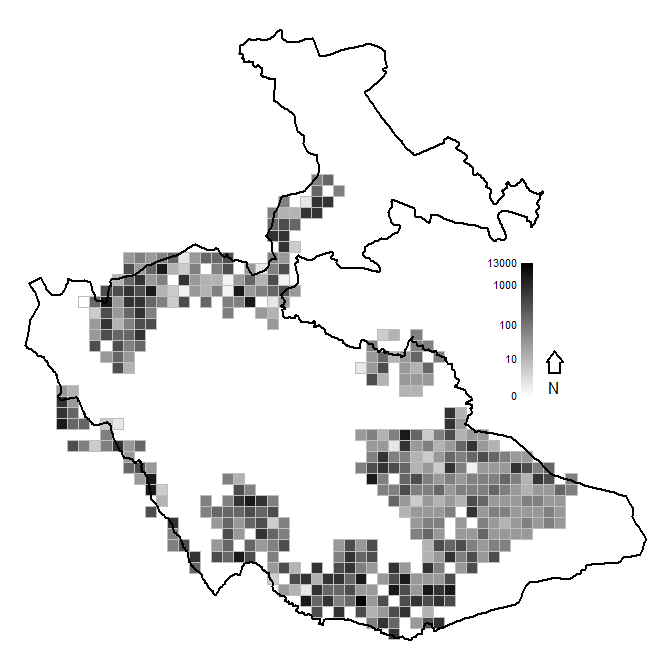


Figure S2. Log-transformed availability of herbaceous gorilla food species (i.e. energy density in kcal/m^2^ based on herb biomass and nutritional content) per 500 x 500 m grid cell in Bwindi Impenetrable National Park, Uganda. The sampled grid cells are indicated as grey boxes. The shades of grey depict the energy density values: the darker the colour, the higher the herbaceous energy density.

Section S3 Utilization of an area

We used distance travelled as a proxy for the utilization of a chosen area because we could not assess the gorillas’ temporal use when following only their trails, but we assumed a positive relation between the distance travelled and the area used. Using this proxy is justified because mountain gorillas spend about 50% of their day feeding and spend only little time exclusively travelling to search for food [8]. To further examine our assumption that for gorillas there is a positive relation between the distance travelled and the area used (i.e. spatial utilization), we investigated activity patterns of one study group (Ky) using instantaneous scan sampling at five minute intervals of the group’s activity (*N* = 18,459) recorded over the study period. We found that the group travelled in 3.4% of all scans (57.1% feeding, 39.1% resting, 0.4% unknown). The group rarely travelled long distances without feeding because they travelled continuously for at least two consecutive scans (=travel bout) in only 1.4% of all scans. During the travel bouts (range of number of consecutive travel scans: 2-7), the group travelled for an average of 9.35 minutes (range: 5-35 min). Given an average walking speed of 0.5 m/s [7], this corresponds to an average travel distance of 280.5 m per group travel bout (range: 150-1050 m). In 74% of all travelling bouts (*N* = 111), the group travelled continuously for only 5-10 minutes (two consecutive scans), which corresponds to a travel distance of approximately 150-300 m, whereas the gorillas travelled for 30-35 minutes continuously in only 1% of all travel bouts (seven consecutive scans; figure S3).


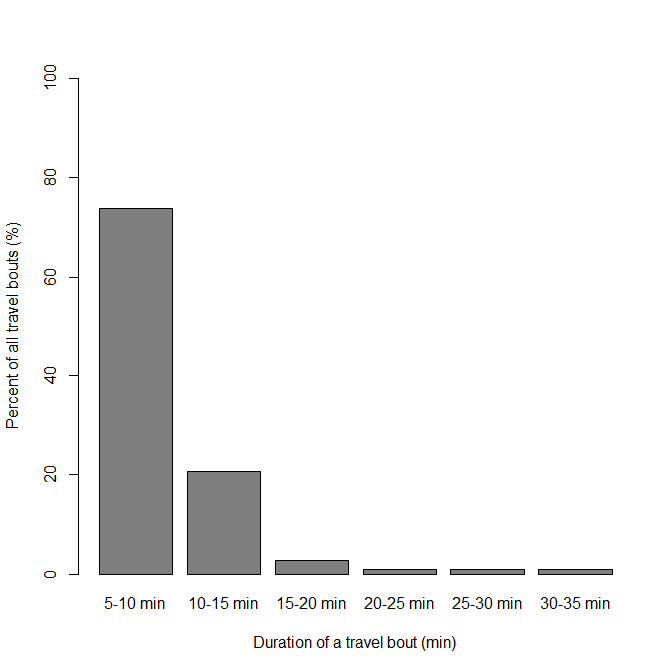
Figure S3. Percent of the various durations of travel bouts in minutes contributing to all travel bouts (instantaneous scans reflecting the behaviour of the entire group Ky during which the group travelled consecutively, *N* = 111). A travel bout consists of at least two scans during which the group travelled consecutively. Note that the group travelled consecutively in in only 1.4% of all scans.

Section S4 Unhabituated groups

In a census that covered all of Bwindi Impenetrable National Park in 2011 (one year before this study), 26 unhabituated gorilla groups were found (mean group size = 7; range: 2-17) [9]. Using the location data from the census, we determined minimum convex polygon (MCP) home ranges for those unhabituated groups in ArcGIS 9.3 (ESRI Inc., Redlands CA, USA) and identified the centroid of points that form the MCP centers applying Hawth’s Tool [10]. Because on average only 3.1 location points (range: 1-11) per group were available, the estimated MCP home ranges are a rough approximation of the unhabituated groups’ ranges. To demonstrate that omitting these groups did not have a major impact on our results, we plotted the centroids of the MCP home ranges of the unhabituated gorilla groups from the 2011 census and the study groups’ home ranges and core areas, respectively (figure S4a and S4b). The centroids of three of the 26 unhabituated groups (group sizes: 3, 9 and 17) were found in the periphery of the 2012-2013 annual home ranges of some study groups (figure S4a) but none in the study groups’ core areas (figure S4b). One unhabituated group’s home range centroid (group size: 3) was found in the periphery of the home ranges of groups Mu and Ru but not within their core areas. The home range centroid of another unhabituated group (group size: 9) was found in the periphery of Kak’s and Bw’s home ranges but not in their core areas. Lastly, the MCP centroid of a third unhabituated group (group size: 17) was found at the periphery of the home ranges of groups Kah and Bu but not within the core areas.


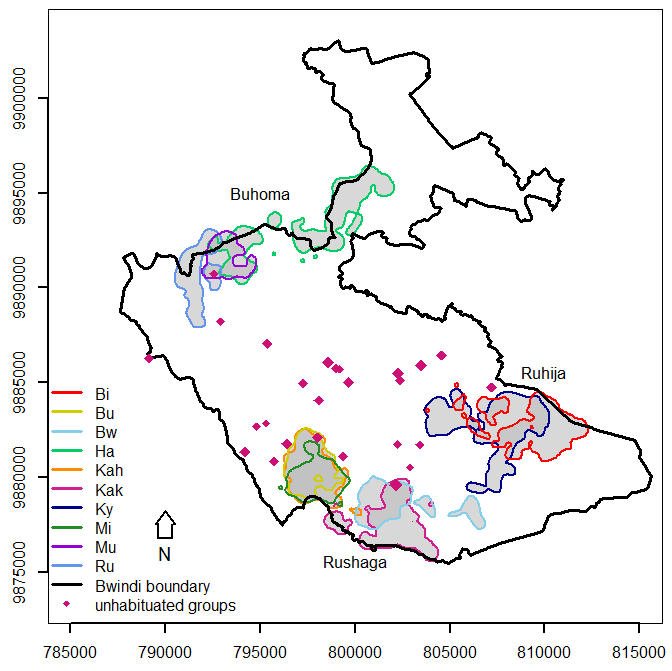


Figure S4a. Location of the annual kernel home ranges of the study groups and the centroids of the MCP home ranges of the 26 unhabituated groups. The areas of the rectangles indicate the sizes of the unhabituated groups (here proportional to the fourth root of group size).


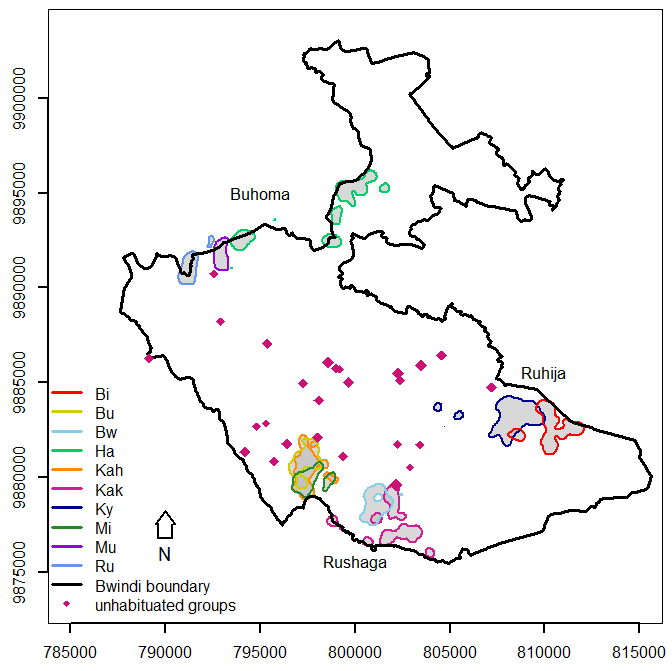


Figure S4b. Location of the annual kernel core areas of the study groups and the centroids of the MCP home ranges of the 26 unhabituated groups. The areas of the rectangles indicate the sizes of the unhabituated groups (here proportional to the fourth root of group size).

Section S5 Determination of the date at which sufficient data on previous use were available

As we did not have any data about previous use by both the groups and their neighbours prior to the start of the study, we plotted both the previous use by the group and by the neighbours against time and calculated moving averages of the predictors for five days and included them into the plots. By visually inspecting these plots, we determined the date at which both the previous use by the group (figure S5a) and by the neighbours (figure S5b) reached a plateau. We determined October 1st 2012 (five months after the start of the data collection) for both the previous use by the group and by the neighbours as appropriate and hence only included data from October 1st 2012 onward into the respective analysis.


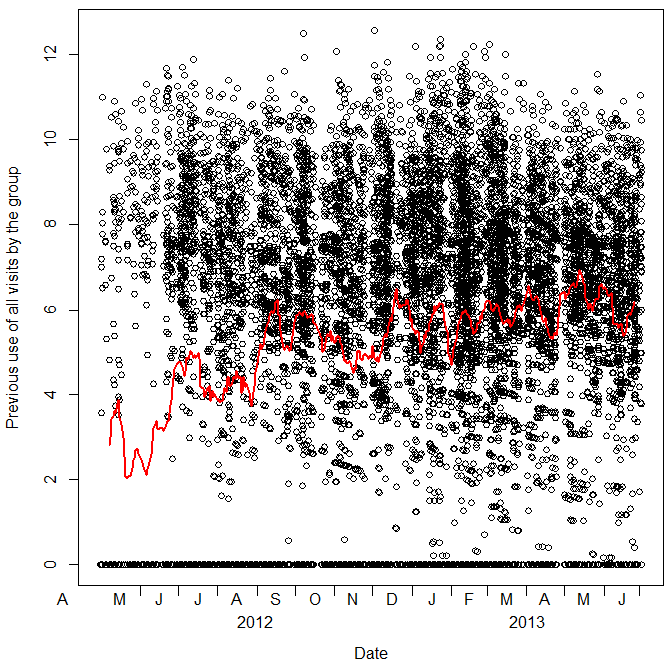


Figure S5a. Determination of the date at which sufficient data on the previous use by the group were available. The previous use by the group is plotted against time. The moving average for five day units of the predictor is shown as a red line.


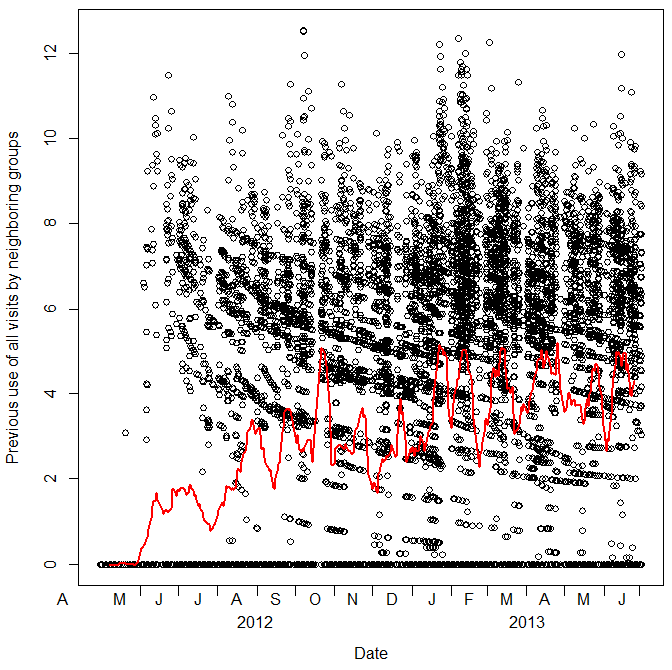


Figure S5b. Determination of the date at which sufficient data on the previous use by neighbouring groups were available. The previous use by neighbouring groups is plotted against time. The moving average for five day units of the predictor is shown as a red line.


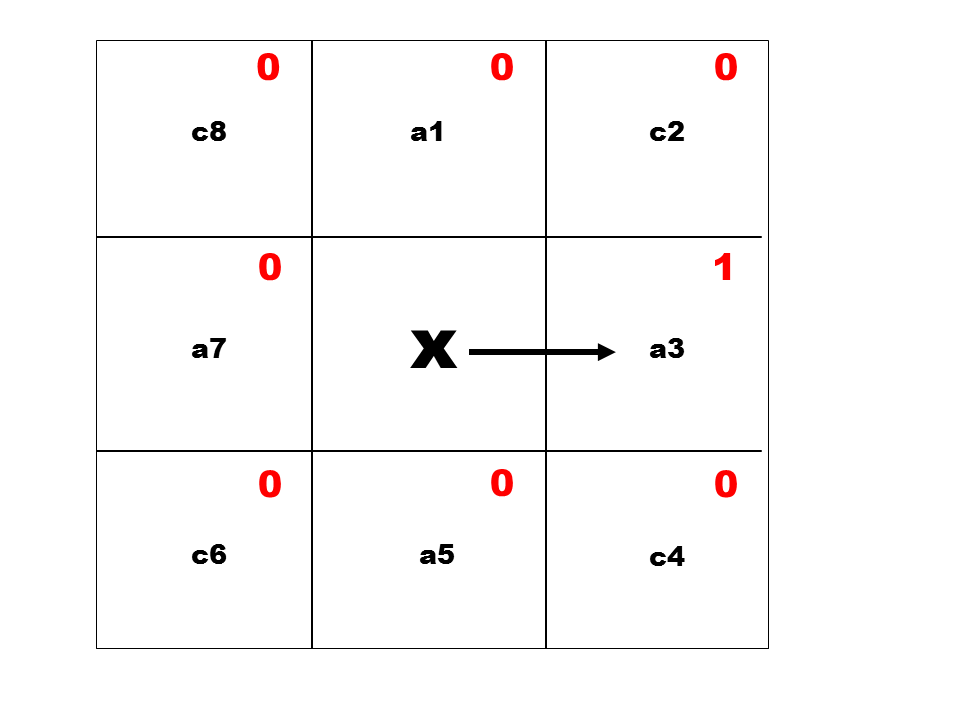


Figure S6. Graphical representation of a group’s decision regarding which of the eight surrounding 500 x 500 m grid cells to choose. *X* marks the location of the group (cell of origin) and *a* and *c* are available grid cells that can be chosen and hence moved to. As the probability that a group would choose an adjacent cell *a* was higher than a cell *c,* bordering only the corner of cell *X*, we adjusted the probabilities of cells to be chosen in the permutation test accordingly (electronic supplementary material S7). The arrow indicates the movement decision of a group to move from grid cell *X* to cell *a3*. Therefore, *a3* was assigned a one as indicated by the red number, whereas the rest of the surrounding cells were each assigned a zero. The permutation test (electronic supplementary material S7) randomly shuffled the assigned one from the actual chosen grid cell *a3* among all surrounding cells *a* and *c*.

Section S7 Statistical analysis

*Details of the model implementation to test the probability of choosing a particular area*

We used a Generalized Linear Mixed Model with binomial error structure and logit link function to test for the effects of herbaceous food availability and both the previous use by the group and by neighbouring groups on the probability that a group would choose a particular area (i.e., grid cell). Besides the test predictors, an offset-term and random effects, we included the random slopes of herbaceous food availability, the previous use by the group and the previous use by neighbouring groups within group ID, grid cell ID, choice ID and both the previous use by the group and by the neighbours within group-grid cell ID [11,12].

We assessed model stability by excluding each level of the random effects one at a time and compared the estimates for each predictor with those obtained for the full data set, which indicated no influential cases (table S7). Using the function vif of the package car [13], we determined Variance Inflation Factors (VIF) [14]. VIFs, determined for a corresponding standard linear model lacking the random effects, did not reveal any collinearity issues among the predictors (maximum VIF = 1.03).

We derived significance (*p* < 0.05) by means of a permutation test [5,15] analogous to that used by Wright and Robbins [16]. More specifically, we permuted the chosen grid cell within the surrounding grid cells for each decision to move into a new grid cell (figure S6). As the probability that the gorillas would choose one of the four directly adjacent cells was higher than the probability that they would choose a cell bordering the corner of the cell of origin (1581 choices to directly adjacent cells and 196 choices to cells bordering the corner of the cell of origin), we adjusted the probabilities for a particular cell to be randomly chosen correspondingly. We conducted a total of 1,000 permutations into which we included the original choices as one permutation. As a measure of the overall effect of the test predictors, we determined the likelihood ratio test statistic (i.e. the chi-square [17]) associated with the comparison of the full model with a null model lacking the test predictors but comprising the same random effects structure and the offset-term as the full model [18]. Significance of the full model was determined as the proportion of χ^2^-values obtained from the permuted choices that were at least as large as the one obtained for the original choices. Correspondingly, significance of the individual test predictors was derived as the proportion of absolute estimates derived for the permuted choices that were at least as large as the one obtained for the original choices. We fitted the model using the function glmer of the lme4 package [19] in R [20].

The R syntax of the fitted model was:

contr=glmerControl(optimizer="nloptwrap", optCtrl=list(maxfun=1000000))

res = glmer (chosen (1/0) ~ herbaceous food availability + previous use by the group + previous use by neighbouring groups + offset(log(1/number of surrounding grid cells)) +

(1+herbaceous food availability+previous use by the group+previous use by neighbouring groups||group ID) +

(1+herbaceous food availability+previous use by the group+previous use by neighbouring groups||grid cell ID) +

(1+herbaceous food availability+previous use by the group+previous use by neighbouring groups||choice ID) +

(1+previous use by the group +previous use by neighbouring groups||group-grid cell ID),

data=data, family=binomial, control=contr)

*Details of the model implementation to test the utilization of a chosen area*

We used a linear mixed model with Gaussian error structure and identity link to test for the effects of herbaceous food availability and both the previous use by the group and by neighbouring groups on the utilization of a chosen area (i.e. distance travelled per grid cell). For each group, the between groups variation of the predictor variables (herbaceous food availability and both the previous use of each grid cell by the group and by neighbours) was quite high and was approximately as high as the variation within groups. In this case, the relation between the utilization of a chosen area and the predictors could potentially differ within and between groups. Therefore, we used the method of ‘within-subjects centring’ [21] by including herbaceous food availability, previous use by the group and previous use by neighbours centred to a mean of zero per group (=within-groups variation) and the mean of this predictor per group (=between-groups variation) in the model. The coefficients then separate the within- and the between-groups effects of the predictors on the utilization of a chosen area [21]. For both the within- and the between-groups effects, we had the same predictions.

We included an autocorrelation term derived analogously to that described by Fürtbauer et al. [22] but based on spatial distance and time lag (standard deviations of the weighting functions for the optimization were determined separately but simultaneously for spatial distance and time lag). To keep error I rate at the nominal level of 5%, we included the random slopes of the within-groups effects of herbaceous food availability, previous use by the group, previous use by neighbouring groups and the autocorrelation term within group ID as well as the within-groups effects of previous use by the group within grid cell ID and group-grid cell ID [11,12].

We visually inspected qqplots and the residuals plotted against fitted values to check for the assumption of normally distributed and homogenous residuals and found no violations. VIFs [14], determined for a model lacking the random effects, indicated no collinearity issue (maximum VIF = 1.43). An investigation of model stability (see above) found no influential levels of random effects (table S7). Using a likelihood ratio test, we compared the full model to the corresponding null model only comprising the random effects and the autocorrelation term [18]. Only after establishing significance of the full model compared to the null model (*p* < 0.05), we considered individual *p*-values based on likelihood ratio tests comparing the full model with a reduced model excluding the respective predictor one at a time [12]. We fitted the model using the function lmer of the lme4 package [19].

The R syntax of the fitted model was:

res = lmer (log-transformed distance travelled per grid cell ~ within-groups effect of herbaceous food availability + between-groups effect of herbaceous food availability + within-groups effect of previous use by the group + between-groups effect of previous use by the group + within-groups effect of previous use by neighbouring groups + between-groups effect of previous use by neighbouring groups + autocorrelation term +

(1+within-groups effect of herbaceous food availability+within-groups effect of previous use by the group+within-groups effect of previous use by neighbouring groups+autocorrelation term||group ID) +

(1+within-groups effect of previous use by the group||grid cell ID) +

(1+within-groups effect of previous use by the group||group-grid cell ID),

data=data, REML=F)

Table S7. Model stability of the permutation test and the mixed model results investigating the factors influencing the probability that Bwindi mountain gorilla groups would choose a particular area (i.e. grid cell) and the utilization of a chosen area (quantified as distance travelled per grid cell). For each model we show the estimates (Est) derived for the full data set and the minimum (Min) and the maximum (Max) of the estimates derived when excluding levels of random effects one at a time. The autocorrelation term (Autocor) represents temporal and spatial autocorrelation. Empty cells indicate variables not included in a model.

| Response variable | Probability of choosing a particular area | | | Utilization of a chosen area | | |
| --- | --- | --- | --- | --- | --- | --- |
| Predictor variable | Est | Min | Max | Est | Min | Max |
| Intercept | 0.072 | 0.053 | 0.102 | 5.970 | 5.931 | 5.993 |
| Herbaceous food availability (within) | ^a^ | | | 0.000 | -0.019 | 0.018 |
| Herbaceous food availability (between) | 0.080 | 0.040 | 0.092 | -0.040 | -0.067 | -0.007 |
| Previous use by the group (within) | ^a^ | | | 0.084 | 0.067 | 0.098 |
| Previous use by the group (between) | 0.354 | 0.304 | 0.393 | 0.076 | 0.045 | 0.122 |
| Previous use by the neighbours (within) | ^a^ | | | -0.032 | -0.067 | -0.007 |
| Previous use by the neighbours (between) | 0.002 | -0.026 | 0.024 | -0.161 | -0.181 | -0.133 |
| Autocor |  | | | 0.255 | 0.230 | 0.290 |

^a^There were no within-groups effects for this model.

S8 Sensitivity analyses to test the robustness of the chosen bandwidth *h*

To ensure the robustness of the chosen parameter (bandwidth fixed to *h* = 200) to determine home range estimates, we conducted a sensitivity analysis by randomly sampling 25%, 50% and 75% of the location data used to estimate the annual home range and core area size for each group (i.e. one location point per day and group). Using the subsamples, we determined the home range and core area sizes and compared them to the home range size estimates derived for the full data set (i.e. one location point per day and group) [23]. The analyses revealed the chosen parameter (bandwidth fixed to *h* = 200) provided rather robust results (figure S8).


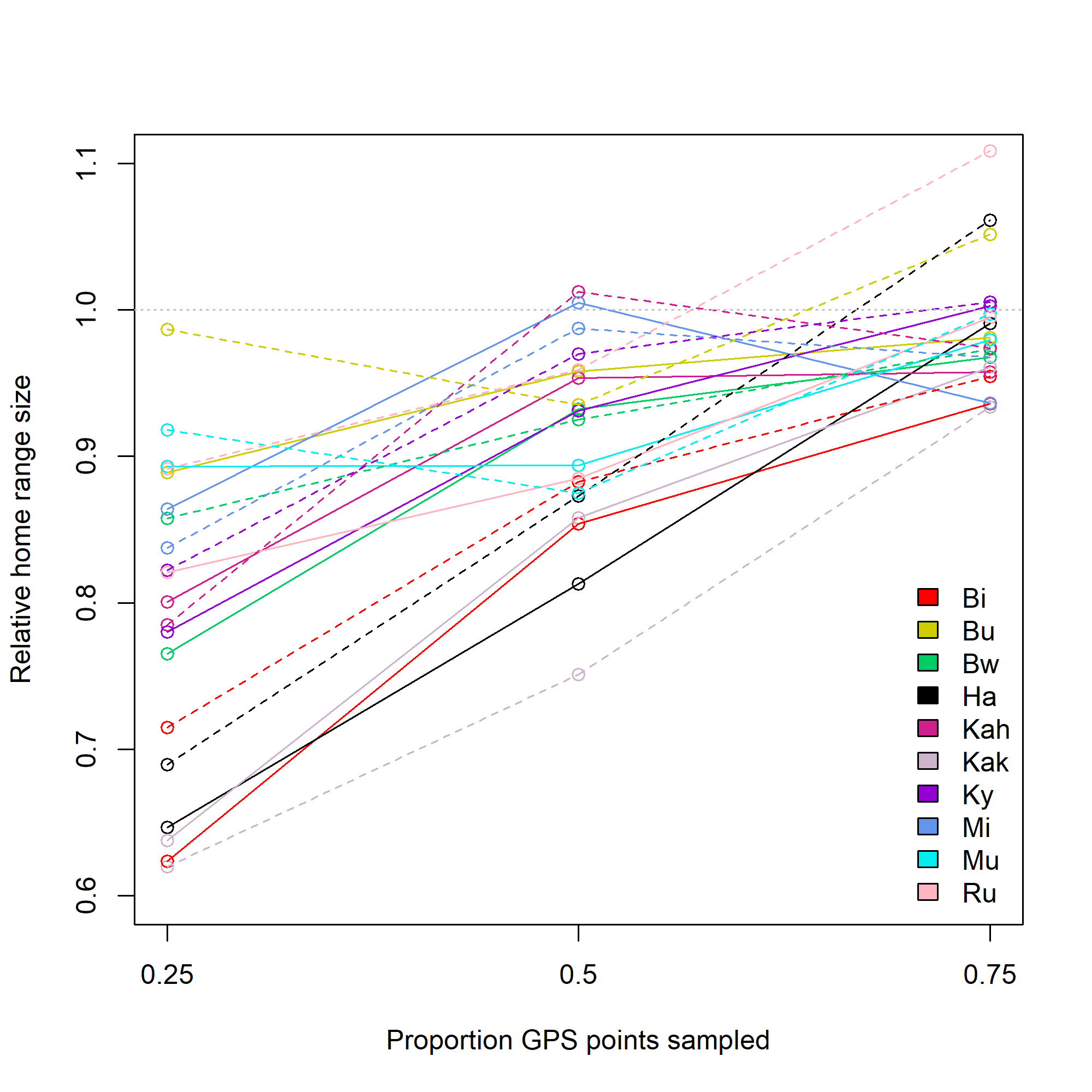


Figure S8. Sensitivity analyses to ensure robustness of our home range size estimates using the chosen parameter (bandwidth *h* = 200). We calculated the ratio between the home range sizes estimated using the subsets (25%, 50% and 75% of full data set) and the home range sizes obtained when using the full data set (i.e. one location point per day and group). The y-axis shows the ratio between the home range size obtained from a given subset and the home range size obtained for the full data. The x-axis shows the proportion of sampled GPS points. Dashed and solid lines indicate the 50% and 90% kernel home range size estimates, respectively, and different colours indicate different groups.

Section S9 Proportions of dyadic annual home range and core area overlap

Using the kernel density home range estimates, we calculated the dyadic proportion of the annual home range of one group (=focal) *HR_i,j_* covered by the home range of a neighbouring group as follows:

*HR_i,j_= A_i,j_ / A_i_*,

where *A_i,j_* is the area of the intersection between the two home ranges of groups *i* and *j*, and *A_i_* is the area of the home range of the focal group *i*. Values ranged from zero to one, with zero indicating no overlap and one indicating a complete overlap of two home ranges. We only included dyads whose annual 90% kernel home range overlap estimate was greater than zero (table S9). We used the adehabitatHR package [24] in R [20] for this analysis.

Table S9. Proportion of annual home range (90% kernel home range) and core area (50% kernel home range) overlap for the 20 group dyads of Bwindi mountain gorillas. Overlap estimates range from zero (=no overlap) to one (=100% overlap).

| Focal | Neighbour | Group dyad | Annual home range overlap | Annual core area overlap |
| --- | --- | --- | --- | --- |
| Bi | Ky | Bi_Ky | 0.44 | 0.07 |
| Ky | Bi | Ky_Bi | 0.35 | 0.06 |
| Bu | Mi | Bu_Mi | 0.64 | 0.21 |
| Mi | Bu | Mi_Bu | 0.71 | 0.25 |
| Bu | Kah | Bu_Kah | 0.92 | 0.73 |
| Kah | Bu | Kah_Bu | 0.74 | 0.54 |
| Bw | Kak | Bw_Kak | 0.40 | 0.17 |
| Kak | Bw | Kak_Bw | 0.31 | 0.11 |
| Bw | Kah | Bw_Kah | 0.01 | 0 |
| Kah | Bw | Kah_Bw | 0.01 | 0 |
| Mi | Kah | Mi_Kah | 0.92 | 0.69 |
| Kah | Mi | Kah_Mi | 0.66 | 0.42 |
| Kah | Kak | Kah_Kak | 0.01 | 0 |
| Kak | Kah | Kak_Kah | 0.01 | 0 |
| Mu | Ha | Mu_Ha | 0.54 | 0 |
| Ha | Mu | Ha_Mu | 0.17 | 0 |
| Mu | Ru | Mu_Ru | 0.25 | 0 |
| Ru | Mu | Ru_Mu | 0.18 | 0 |
| Ha | Ru | Ha_Ru | 0.02 | 0 |
| Ru | Ha | Ru_Ha | 0.05 | 0 |

Table S10. Random effects for the permutation test and the mixed model results investigating the factors influencing the probability that Bwindi mountain gorilla groups would choose a particular area (i.e., grid cell) and the utilization of a chosen area (quantified as distance travelled per grid cell).

| Probability of choosing a particular area | | |
| --- | --- | --- |
| Random effect | Term^a^ | Standard deviation |
| Group ID | Intercept | 0.038 |
| Group ID | Herbaceous food availability | 0.067 |
| Group ID | Previous use by the group | 0.151 |
| Group ID | Previous use by the neighbours | 0.064 |
| Grid cell ID | Intercept | 0.190 |
| Grid cell ID | Herbaceous food availability | <0.001 |
| Grid cell ID | Previous use by the group | <0.001 |
| Grid cell ID | Previous use by the neighbours | 0.145 |
| Choice ID | Intercept | <0.001 |
| Choice ID | Herbaceous food availability | <0.001 |
| Choice ID | Previous use by the group | <0.001 |
| Choice ID | Previous use by the neighbours | <0.001 |
| Group-grid cell ID | Intercept | <0.001 |
| Group-grid cell ID | Previous use by the group | <0.001 |
| Group-grid cell ID | Previous use by the neighbours | <0.001 |
| Utilization of a chosen area | | |
| Random effect | Term^a^ | Standard deviation |
| Group ID | Intercept | 0.046 |
| Group ID | Herbaceous food availability (within) | <0.001 |
| Group ID | Previous use by the group (within) | 0.069 |
| Group ID | Previous use by the neighbours (within) | 0.082 |
| Group ID | Autocorrelation | 0.125 |
| Grid cell ID | Intercept | 0.132 |
| Grid cell ID | Previous use by the group (within) | 0.135 |
| Group-grid cell ID | Intercept | 0.114 |
| Group-grid cell ID | Previous use by the group (within) | <0.001 |

^a^Intercept indicates a random intercept, other entries indicate a random slope of the indicated predictor within the random effect shown to its left.

Table S11. Mean and standard deviation (SD) of the original values of the predictor variables testing the probability that Bwindi mountain gorilla groups would choose a particular area (i.e., grid cell) and the utilization of a chosen area (quantified as distance travelled per grid cell).

| Response variable | Probability of choosing a particular area | | Utilization of a chosen area | |
| --- | --- | --- | --- | --- |
| Predictor variable | Mean | SD | Mean | SD |
| Herbaceous food availability | 1047.965 | 1783.97 | 1208.56 | 1973.75 |
| Herbaceous food availability (within)^b^ | ^a^ | | 0.00 | 1.71 |
| Herbaceous food availability (between)^b^ | ^a^ | | 5.84 | 0.56 |
| Previous use by the group | 4788.73 | 14030.96 | 9671.88 | 21927.55 |
| Previous use by the group (within)^b^ | ^a^ | | 0.00 | 2.73 |
| Previous use by the group (between)^b^ | ^a^ | | 7.25 | 0.90 |
| Previous use by the neighbours | 1661.242 | 9887.16 | 1956.14 | 8847.35 |
| Previous use by the neighbours (within)^b^ | ^a^ | | 0.00 | 2.71 |
| Previous use by the neighbours (between)^b^ | ^a^ | | 3.78 | 2.19 |

^a^not applicable

^b^log-transformed

Section S12 References for Supplementary Material

1. Ganas J, Nkurunungi JB, Robbins MM. 2009 A preliminary study of the temporal and spatial biomass patterns of herbaceous vegetation consumed by mountain gorillas in an afromontane rain forest. *Biotropica* **41**, 37–46. (doi:10.1111/j.1744-7429.2008.00455.x)

2. Ganas J, Robbins MM. 2005 Ranging behavior of the mountain gorillas (*Gorilla beringei beringei*) in Bwindi Impenetrable National Park, Uganda: a test of the ecological constraints model. *Behav. Ecol. Sociobiol.* **58**, 277–288. (doi:10.1007/s00265-005-0920-z)

3. Ganas J, Ortmann S, Robbins MM. 2008 Food preferences of wild mountain gorillas. *Am. J. Primatol.* **70**, 927–938. (doi:10.1002/ajp.20584)

4. Zar JH. 1999 *Biostatistical analysis.* Upper Saddle River, New Jersey: Prentice Hall.

5. Manly BFJ. 1997 *Randomization, bootstrap and Monte Carlo methods in biology.* London, UK: Chapman & Hall.

6. Ganas J, Ortmann S, Robbins MM. 2009 Food choices of the mountain gorilla in Bwindi Impenetrable National Park, Uganda: the influence of nutrients, phenolics and availability. *J. Trop. Ecol.* **25**, 123–134. (doi:10.1017/S0266467408005701)

7. Wright E, Robbins AM, Robbins MM. 2014 Dominance rank differences in the energy intake and expenditure of female Bwindi mountain gorillas. *Behav. Ecol. Sociobiol.* **68**, 957–970. (doi:10.1007/s00265-014-1708-9)

8. Watts DP. 1988 Environmental influences on mountain gorilla time budgets. *Am. J. Primatol.* **15**, 195–211.

9. Roy J *et al.* 2014 Challenges in the use of genetic mark-recapture to estimate the population size of Bwindi mountain gorillas (*Gorilla beringei beringei*). *Biol. Conserv.* **180**, 249–261. (doi:10.1016/j.biocon.2014.10.011)

10. Beyer HL. 2004 *Hawth’s Analysis Tools for ArcGIS*.

11. Schielzeth H, Forstmeier W. 2009 Conclusions beyond support: overconfident estimates in mixed models. *Behav. Ecol.* **20**, 416–420. (doi:10.1093/beheco/arn145)

12. Barr DJ, Levy R, Scheepers C, Tily HJ. 2013 Random effects structure for confirmatory hypothesis testing: Keep it maximal. *J. Mem. Lang.* **68**, 255–278. (doi:10.1016/j.jml.2012.11.001)

13. Fox J, Weisberg S. 2011 *An R Companion to Applied Regression.* Thousand Oaks: Sage Publications.

14. Field A. 2005 *Discovering Statistics using SPSS.* London, UK: Sage Publications.

15. Adams DC, Anthony CD. 1996 Using randomization techniques to analyse behavioural data. *Anim. Behav.* **51**, 733–738.

16. Wright E, Robbins MM. 2014 Proximate mechanisms of contest competition among female Bwindi mountain gorillas (*Gorilla beringei beringei*). *Behav. Ecol. Sociobiol.* **68**, 1785–1797. (doi:10.1007/s00265-014-1788-6)

17. Dobson AJ. 2002 *An Introduction to Generalized Linear Models.* Boca Raton: Chapman & Hall/CRC.

18. Forstmeier W, Schielzeth H. 2011 Cryptic multiple hypotheses testing in linear models: overestimated effect sizes and the winner’s curse. *Behav. Ecol. Sociobiol.* **65**, 47–55. (doi:10.1007/s00265-010-1038-5)

19. Bates D, Mächler M, Bolker B, Walker S. 2015 Fitting linear mixed-effects models using lme4. *J. Stat. Softw.* **67**, 1–48. (doi:10.18637/jss.v067.i01)

20. R Core Team. 2015 *R: A language and Environment for Statistical Computing*. Vienna, Austria: R Foundation for Statistical Computing. See http://www.R-project.org.

21. van de Pol M, Wright J. 2009 A simple method for distinguishing within- versus between-subject effects using mixed models. *Anim. Behav.* **77**, 753–758. (doi:10.1016/j.anbehav.2008.11.006)

22. Fürtbauer I, Mundry R, Heistermann M, Schülke O, Ostner J. 2011 You mate, I mate: Macaque females synchronize sex not cycles. *PLoS ONE* **6**, e26144. (doi:10.1371/journal.pone.0026144)

23. Caillaud D, Ndagijimana F, Giarrusso AJ, Vecellio V, Stoinski TS. 2014 Mountain gorilla ranging patterns: Influence of group size and group dynamics. *Am. J. Primatol.* **76**, 730–746. (doi:10.1002/ajp.22265)

24. Calenge C. 2006 The package ‘adehabitat’ for the R software: A tool for the analysis of space and habitat use by animals. *Ecol. Model.* **197**, 516–519. (doi:10.1016/j.ecolmodel.2006.03.017)
